# Optimizing low-dose rituximab protocol for ABO-mismatched kidney transplantation: long-term outcomes in a single-center retrospective cohort study

**DOI:** 10.1007/s10157-026-02882-1

**Published:** 2026-05-26

**Authors:** Tomoaki Yamanoi, Takanori Sekito, Moto Tokunaga, Ichiro Tsuboi, Kasumi Yoshinaga, Yuki Maruyama, Tatsushi Kawada, Risa Kubota, Yusuke Tominaga, Takuya Sadahira, Satoshi Katayama, Takehiro Iwata, Shingo Nishimura, Kensuke Bekku, Yasuhiro Onishi, Hidemi Takeuchi, Katsuyuki Tanabe, Hiroshi Morinaga, Koichiro Wada, Motoo Araki

**Affiliations:** 1 Department of Urology, Okayama University Graduate School of Medicine, Dentistry and Pharmaceutical Sciences, 2-5-1, Shikata-cho, Kita-ku, Okayama, 700-8558 Japan; 2 Department of Inflammation and Immunity, Lerner Research Institute, Cleveland Clinic, Cleveland, OH USA; 3 Department of Urology, NHO Okayama Medical Center, Okayama, Japan; 4 Department of Nephrology, Rheumatology, Endocrinology and Metabolism, Okayama University Graduate School of Medicine, Dentistry and Pharmaceutical Sciences, Okayama, Japan; 5 Department of Urology, Shimane University Faculty of Medicine, Shimane, Japan

**Keywords:** Kidney transplantation, ABO-mismatch, Low-dose rituximab, Graft survival, Passenger lymphocyte syndrome

## Abstract

**Background:**

ABO-mismatched kidney transplantation (KTx) expands donor availability but increases risks of antibody-mediated rejection and passenger lymphocyte syndrome (PLS). While rituximab (Rit) potentially mitigates these complications, conventional high-dose regimens (375 mg/m^2^) elevate infectious and hematologic toxicity. We implemented low-dose Rit induction (200 mg/body) for desensitization in minor/major ABO-mismatched and DSA-positive KTx, evaluating its efficacy and safety over 15-years.

**Methods:**

This single-center retrospective cohort (May 2009–April 2024) analyzed 161 adult KTx recipients: Rit (n = 107) and Non-Rit (n = 54) groups. All received tacrolimus, mycophenolate mofetil, prednisolone, and basiliximab; high-risk patients also underwent plasmapheresis. Outcomes included graft survival, biopsy-proven acute rejection, de novo donor-specific antibody (DSA) formation, infection, severe neutropenia, and PLS.

**Results:**

1-year graft survival was 100% in both groups. 5-year death-censored graft survival was 95.8% (Rit) vs 95.9% (Non-Rit), respectively (log-rank *P* = 0.43). Biopsy-proven acute rejection (7.5% vs 3.7%, *P* = 0.50) and de novo DSA production were equivalent (Class I: 5.5% vs 2.2%; Class II: 6.6% vs 8.7%; both *P* = 1.00), with lower mean fluorescent intensity (MFI) in the Rit group. Cytomegalovirus disease, urinary tract infection and fungal infection rates were comparable between both groups. Grade 4 neutropenia was not associated with Rit (OR 2.65; 95% CI 0.63–11.0; *P* = 0.18). Blood transfusion for hemoglobin declines occurred in 5.6% vs 7.4%, with preserved haptoglobin in all cases, indicating no PLS.

**Conclusions:**

Low-dose Rit induction achieves excellent graft survival and effective PLS prevention, without increasing toxicity, supporting its adoption as an optimal desensitization strategy.

**Supplementary Information:**

The online version contains supplementary material available at 10.1007/s10157-026-02882-1.

## Introduction

Kidney transplantation (KTx) represents the optimal treatment for end-stage kidney disease (ESKD), offering superior quality of life and survival compared to dialysis [1, 2]. However, the persistent shortage of compatible donor organs—particularly acute in Japan, where deceased donor transplantation rates significantly lag Western countries—has necessitated the expansion of living-donor criteria to include ABO-incompatible and human leukocyte antigen antibody-incompatible (HLAi) KTx [3]. These immunologically challenging procedures require sophisticated desensitization strategies to overcome preformed antibodies and prevent antibody-mediated rejection [4, 5].

Rituximab (Rit), a chimeric anti-CD20 monoclonal antibody, selectively depletes B lymphocytes and has become an integral part of desensitization protocols. Standard dosing (375 mg/m^2^) effectively reduces rejection and donor-specific antibody (DSA) formation but increases infection and hematologic toxicity [6–10]. While recent studies suggest that low-dose Rit may preserve efficacy while reducing these adverse effects, large-cohort evidence remains limited [10–15]. Notably, both standard-dose and low-dose rituximab-based protocols have demonstrated safety and efficacy in Japanese multicenter and single-center studies of ABO-incompatible recipients, supporting the feasibility of individualized dosing strategies [6, 9].

Within the spectrum of ABO compatibility, minor ABO-mismatched KTx (a pair of different ABO blood types without donor-derived anti-recipient antibodies) presents unique immunological considerations. While generally considered immunologically compatible, this type of transplantation carries a risk of passenger lymphocyte syndrome (PLS), a potentially severe complication where donor-derived B lymphocytes produce antibodies against recipient erythrocytes, leading to hemolysis. PLS incidence varies dramatically across organ types, ranging from 9–20% in KTx to 30–40% in liver transplants and up to 70% in cardiothoracic procedures, reflecting differences in graft lymphoid content and immunosuppressive intensity [16–20]. While generally considered immunologically compatible, minor ABO-mismatched KTx carries sufficient PLS risk to warrant prophylactic intervention. Traditional PLS prevention strategies, including graft irradiation and intensified immunosuppression, carry substantial morbidity. Graft irradiation may compromise transplant function and elevate malignancy risk, and high-dose immunosuppression often fails to provide selective B-cell targeting [16, 17, 21]. Low-dose Rit may offer an attractive alternative by achieving preemptive B-cell depletion without the complications associated with more invasive approaches [22–24].

Our institution has implemented a standardized low-dose Rit protocol (200 mg/body) across immunologically high-risk recipients, including those with minor/major ABO-mismatches, HLAi conditions, and focal segmental glomerulosclerosis (FSGS) cohorts, over 15 years. We hypothesized that low-dose Rit would achieve comparable or superior transplant outcomes while maintaining a favorable safety profile, thereby establishing a new standard of care for immunologically challenging KTx. Comprehensive evaluation of PLS prevention and safety profile attribution in low-dose Rit protocols remains limited. This study addresses these gaps in immunologically high-risk KTx.

## Materials and methods

### Study design and patient population

We conducted a retrospective cohort study of 161 KTx recipients at Okayama University Hospital between May 2009 and April 2024. Exclusion criteria were as follows: age < 18 years [n = 5], high dose Rit (500 mg/body) [n = 3], and previous graft irradiation [n = 1]).

HLA typing at loci A, B, C, DR, and DQ was performed by the Luminex 200 system (Luminex, Inc., Austin, Texas, United States). Alloantibody binding was measured by the LAB Screen single antigen bead assay (Luminex, Inc.). Beads with a mean fluorescent intensity (MFI) > 500 were considered positive for DSA. This sensitive cutoff was consistently applied throughout the 15-year study period to meticulously detect low-level sensitization. Rit eligibility criteria for this study are as follows: Major ABO-mismatched (recipient has naturally occurring anti-A and/or anti-B antibodies directed against donor ABO antigens), or minor ABO-mismatched (a pair of different ABO blood types without donor-derived anti-recipient antibodies), or positive for DSA, or with FSGS. Rit was administered at a dose of 200 mg/body 1–2 weeks before transplantation. The study population was divided into two groups based on Rit administration: Rit group (n = 107) and Non-Rit group (n = 54).

The study was approved by the Okayama University Institutional Review Board (registration number: 1911—030) and complied with the Declaration of Helsinki and current ethical guidelines. All participants provided written informed consent prior to enrollment in the study.

### Immunosuppression and desensitization protocols

All recipients received standardized triple maintenance immunosuppression consisting of tacrolimus (target 8—12 ng/mL initially, then 5 ng/mL), mycophenolate mofetil (MMF) (20—30 mg/kg), and prednisolone, supplemented with basiliximab induction therapy. Risk-specific modifications were the application of Rit and plasmapheresis as follows:

1. ABO-matched received standard triple immunosuppression plus basiliximab, without Rit.
2. Minor ABO-mismatched received standard triple immunosuppression plus basiliximab, with low-dose Rit administered 1 week before surgery. Importantly, no plasmapheresis was performed in this group unless indicated for other reasons.
3. Major ABO-mismatched received standard triple immunosuppression plus basiliximab, with low-dose Rit administered 1—2 weeks before surgery, and 2—3 sessions of plasmapheresis (double filtration plasmapheresis [DFPP] or plasma exchange [PEX]).
4. Recipients with DSA or FSGS received low-dose Rit administered 1—2 weeks before surgery, regardless of their ABO compatibility status, with plasmapheresis added according to HLA typing/titer and immunological risk assessment.

Since 2019, recipients with a positive DSA titer (MFI ≥ 1000), regardless of the complement-dependent cytotoxicity crossmatch and flow-cytometric crossmatch, have received intravenous immunoglobulin (IVIG) (1—4 g/kg) for 7 days before transplant. Details are shown in Supplementary Figure S1—5.

### Postoperative infection monitoring

Cytomegalovirus (CMV) viremia was defined as pp65 antigenemia assay (≥ 5 positive cells), as quantitative polymerase chain reaction was not available during the study period. CMV disease required the presence of compatible clinical symptoms, including fever ≥ 38 °C or gastrointestinal manifestations. Treatment with oral valganciclovir prophylaxis for CMV viremia and intravenous ganciclovir for persistent antigenemia or CMV disease was initiated with concurrent MMF dose reduction. Other infectious complications were monitored throughout the follow-up period. Urinary tract infection (UTI) was defined as fever ≥ 38 °C with bacteriuria (white blood-cell count ≧5 /high-power fields). Fungal infection was defined as clinically documented infection requiring invasive intervention or systemic antifungal therapy.

### Neutropenia assessment and management

Neutropenia monitoring was part of the routine hematological surveillance following KTx. A complete blood count with differential was obtained at baseline, weekly during the first month post-transplant, and then at regular intervals throughout the follow-up period. Neutropenia severity was graded according to the National Cancer Institute’s Common Terminology Criteria for Adverse Events (CTCAE) version 5.0. Primary neutropenia endpoints included the incidence of severe neutropenia (Grade 3; neutrophil count < 1000 /μL and Grade 4; neutrophil count < 500 /μL) occurring within 1 year post-transplant. In case of G3 neutropenia, granulocyte colony-stimulating factor (G-CSF) was administered regardless of the presence of fever.

### Clinical and laboratory monitoring

All patients underwent regular clinical and laboratory monitoring. Hemolytic parameters, including hemoglobin, lactate dehydrogenase (LDH), and total bilirubin (T-bil) were particularly monitored at the first postoperative week, with subsequent assessments at 2 weeks, 1 month, and 3 months post-transplant. Haptoglobin levels were additionally measured when hemoglobin decline was observed. PLS was defined based on previously published criteria and institutional reference values as meeting the following 4 criteria more than 1 week post-KTx: 1) Blood transfusion due to hemoglobin decline, 2) LDH ≥ 1.5 × upper limit of normal, 3) T-bil ≥ 1.5 mg/dL with indirect bilirubin dominance, and 4) haptoglobin level < 22 mg/dL, measured at the discretion of the attending physician in cases of hemoglobin decline [14, 20]. Direct antiglobulin testing (DAT) was not performed as a routine protocol but was conducted specifically when a postoperative hemoglobin decline was observed or hemolysis was clinically suspected. Peripheral blood lymphocyte subsets were quantified using flow cytometry according to standard clinical protocols, with a normal reference range of 5—24%. Measurements were performed at four days post-Rit and 1 year post-transplant. Protocol biopsies were routinely performed at 2—3 weeks and 1 year post-transplant. Additionally, episode biopsies were conducted based on clinical indications, such as unexplained creatinine elevation > 0.3 mg/dL from baseline or persistent proteinuria and microscopic hematuria.

### Outcomes

The primary outcomes included overall graft/patient survival and death-censored graft survival rates between the Rit and the Non-Rit groups. Secondary outcomes encompassed biopsy-proven acute rejection episodes, clinical infection within 1 year, severe neutropenia within 1 year, and evidence of PLS based on laboratory criteria.

### Statistical analysis

Statistical analyses were conducted using Fisher’s exact test for categorical variables and the Mann–Whitney U test for continuous data, as appropriate. *P*-values < 0.05 were considered statistically significant. Survival curves were estimated by the Kaplan–Meier method and compared with the log-rank test. Logistic regression was applied to determine independent predictors of Grade 4 neutropenia, incorporating recipient demographics, immunological risk factors, baseline clinical characteristics, and treatment-related factors. All analyses were performed using EZR version 1.64 (Saitama Medical Center, Jichi Medical University), a graphical user interface for R (The R Foundation for Statistical Computing, Vienna, Austria), and GraphPad Prism version 6.00 (GraphPad Software, La Jolla, California, USA) [25].

## Results

### Baseline characteristics

Baseline characteristics were comparable between the Rit and the Non-Rit groups, with no significant differences in recipient demographics, cause of ESKD, history of dialysis, or comorbidities (Table 1). All deceased-donor transplants occurred in the Non-Rit group (18.5% vs. 0% in the Rit group; *P* < 0.001). ABO compatibility, HLA antibody status, and desensitization measures differed markedly between groups (Table 2). Initial MMF dosing tended to be slightly higher in the Rit group, and 73.8% underwent pre-transplant plasmapheresis versus none in the Non-Rit group (*P* < 0.001). Preoperative B-lymphocyte counts were effectively depleted in the Rit group (median 0% vs. 11%; *P* < 0.001).

**Table 1:** Baseline demographic and clinical characteristics

Variables	Total (161)	Non-Rit (54)	Rit (107)	*P-value*
Age (y), median (IQR)	48 (35—58)	48 (38—58)	47 (34—58)	0.80
Gender (female), n (%)	49 (30.4)	15 (27.8)	34 (31.8)	0.72
BMI, kg/m^2^, median (IQR)	22.0 (19.1—24.5)	21.8 (19.3—23.7)	22.2 (19.1—25.7)	0.67
Deceased donor, n (%)	10 (6.2)	10 (13.3)	0	< 0.001
Donor age (y), median (IQR)	60 (53—66)	62 (52—67)	59 (54—66)	0.45
CMV high risk (D + R-), n (%)	27 (16.8)	8 (17.0)	19 (17.8)	1.00
HTN, n (%)	99 (61.5)	33 (61.1)	66 (61.7)	1.00
DM, n (%)	33 (20.5)	9 (16.7)	24 (22.4)	0.54
Primary disease, n (%)				0.48
DM nephropathy	27 (16.8)	7 (13.2)	20 (18.7)	
Nephrosclerosis	11 (6.8)	4 (7.5)	7 (6.5)	
CGN	53 (32.9)	17 (31.5)	36 (33.6)	
FSGS	4 (2.5)	0	4 (3.7)	
Other	61 (37.9)	23 (43.4)	38 (35.5)	
Unknown	5 (3.1)	3 (5.7)	2 (1.9)	
PEKT, n (%)	45 (28.0)	12 (22.2)	33 (30.8)	0.27
Dialysis period (m), median (IQR)	11 (0—39)	11 (1—49)	9 (0—32)	0.20

*IQR* Interquartile range, *BMI* Body mass index, *CMV* Cytomegalovirus, *HTN* Hypertension, *DM* Diabetes mellitus, *CGN* Chronic glomerulonephritis, *FSGS* Focal segmental glomerulosclerosis, *PEKT* Preemptive kidney transplantation

**Table 2:** Immunological risk and treatment-related factors

Variables	Total (161)	Non-Rit (54)	Rit (107)	*P-value*
ABO compatibility				< 0.001
Matched, n (%)	74 (45.9)	54 (100)	20 (18.7)	
Minor-mismatched, n (%)	29 (18.0)	0	29 (27.1)	
Major-mismatched, n (%)	58 (36.0)	0	58 (54.2)	
DSA, n (%)				< 0.001
HLA class1 (A, B, C)	15 (9.3)	0	15 (12.1)	
HLA class2 (DR, DQ)	18 (11.1)	0	18 (16.8)	
Initial MMF per kg, median (IQR)	23.0 (19.4—26.5)	21.7 (18.8—25.6)	23.7 (20.1—26.8)	0.07
Plasmapheresis, n (%)	79 (49.1)	0	79 (73.8)	< 0.001
Pre-transplant B-Lymphocyte (%)	0 (0—4)	11 (6—14)	0 (0—0)	< 0.001

*DSA* Donor specific antibody, *HLA* Human leukocyte antigen antibody, *MMF* Mycophenolate mofetil, *IQR* Interquartile range

### Graft survival

**Fig. 1:**
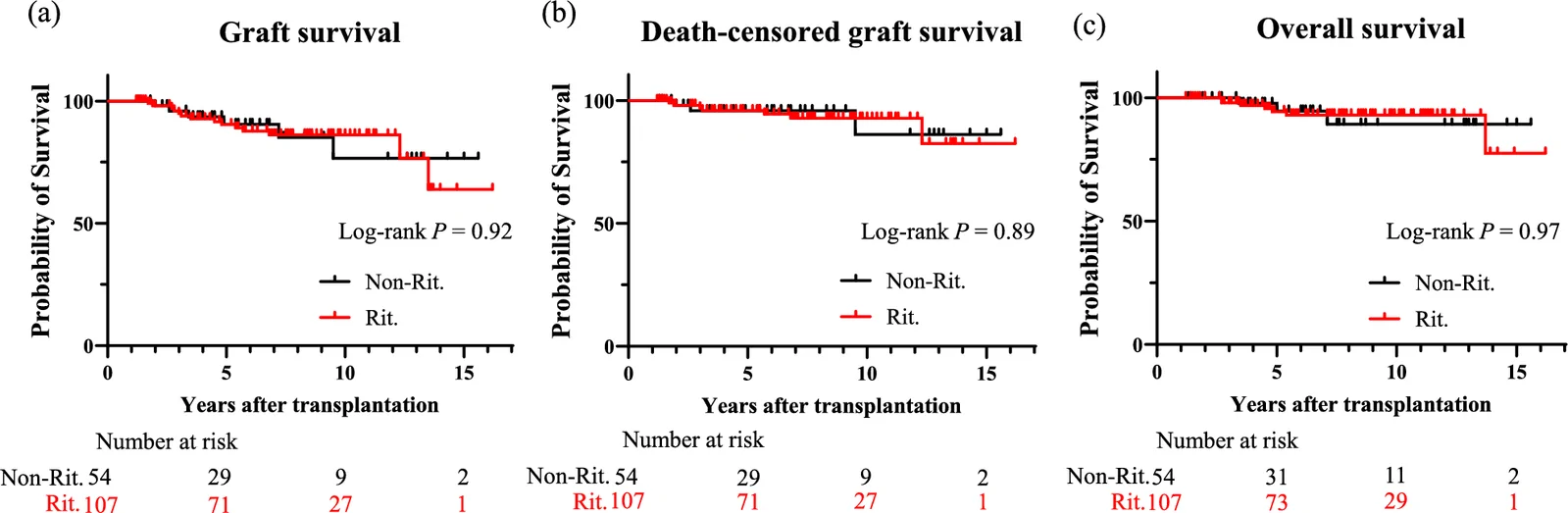
Kaplan–Meier curve of **a** Graft survival, **b** Death-censored graft survival, and **c** Patient survival

At 1 year post-transplant, graft and patient survival rates were 100% in both groups. 5-year graft survival rates were 90.4% in the Non-Rit group and 90.3% in the Rit group (log-rank *P* = 0.92), while 5-year death-censored graft survival rates were 95.8% versus 95.9%, respectively (log-rank *P* = 0.43) (Fig. 1). Patient survival did not differ significantly between groups at 1, 5 or 10 years post-transplant.

### De novo DSA production and biopsy proven acute rejection

Development of de novo DSA was assessed in 137 recipients with available follow-up (Non-Rit: 46 cases, Rit: 91 cases). De novo DSA against HLA Class I antigens (A, B, C) occurred in 2.2% of the Non-Rit vs 5.5% of the Rit group, while Class II DSA (DR, DQ) developed in 8.7% vs 6.6%, respectively (*P* = 1.00) (Table 3). Among groups who developed de novo DSA, the median MFI was numerically higher in the Non-Rit group compared to the Rit group, though this difference was not statistically significant (9069 vs 4324, *P* = 0.39). Biopsy-proven acute rejection rates were not statistically significant between both groups (3.7% vs 7.5%, *P* = 0.50). Furthermore, a subgroup analysis comparing the Non-Rit group (n = 54) with patients receiving Rit solely for minor ABO-mismatch in the absence of preformed DSA or history of FSGS (n = 17) revealed no significant differences in the incidence of de novo DSA (12.5% in the Rit subgroup vs. 10.9% in the Non-Rit group,* P* = 1.00) or biopsy-proven acute rejection (0% vs. 3.7%, *P* = 1.00). Notably, among those who developed de novo DSA, the median MFI tended to be lower in this specific Rit subgroup compared to the Non-Rit group (2,450 vs. 9,069; *P* = 0.25), highlighting the sustained efficacy of low-dose Rit even in minor-mismatched cohorts.

**Table 3:** Postoperative clinical outcome

Variables	Total (161)	Non-Rit (54)	Rit (107)	*P-value*
serum creatinine at 1 year, mg/dL, median (IQR)	1.4 (1.1—1.8)	1.4 (1.0—1.7)	1.4 (1.2—1.8)	0.35
CMV viremia within 1 year, n (%)	59 (36.6)	22 (40.7)	37 (34.6)	0.49
CMV disease within 1 year, n (%)	32 (19.9)	12 (22.2)	20 (18.7)	0.68
Urinary tract infection within 1 year, n (%)	25 (15.5)	6 (11.1)	19 (17.8)	0.36
Fungal infection within 1 year, n (%)	4 (2.5)	0	4 (3.7)	0.30
Grade 3 neutropenia within 1 year, n (%)	56 (34.8)	13 (24.1)	43 (40.2)	0.055
Grade 4 neutropenia within 1 year, n (%)	27 (16.8)	4 (7.4)	23 (21.5)	0.027
B-Lymphocytes at 1 year (%)	2 (0—6)	8 (5—11)	0 (0—2)	< 0.001
de novo DSA, n (%)				1.00
HLA class1 (A, B, C)	6 (4.4)	1 (2.2)	5 (5.5)	
HLA class2 (DR, DQ)	10 (7.3)	4 (8.7)	6 (6.6)	
MFI, arbitrary units (IQR)	5019 (1489—22,054)	9069 (1604—27,867)	4324 (1431—13,388)	0.39
Biopsy-proven acute rejection, n (%)	10 (6.2)	2 (3.7)	8 (7.5)	0.50
Follow period, year, median (IQR)	6.8 (3.9—10.0)	6.1 (3.6—8.5)	7.4 (4.3—10.1)	0.18

*IQR* Interquartile range, *CMV* Cytomegalovirus, Grade 3 neutropenia, neutrophil count < 1000 μL, Grade 4 neutropenia, neutrophil count < 500 μL, *DSA* Donor specific antibody, *HLA* Human leukocyte antigen antibody, *MFI* Mean fluorescent index

### Postoperative infection and neutropenia

CMV viremia and clinical CMV disease within 1 year post-transplant occurred at similar frequencies (Non-Rit vs Rit; 40.7% vs. 34.6%, *P* = 0.49; 22.2% vs 18.7%, *P* = 0.68, respectively). UTI and fungal infection rates were comparable between both groups.

Grade 3 neutropenia occurred in 24.1% of the Non-Rit vs 40.2% of the Rit group (*P* = 0.055), and Grade 4 neutropenia in 7.4% vs 21.5%, respectively (*P* = 0.027) (Table 3). Multivariate logistic regression for Grade 4 neutropenia demonstrated that anti-CMV treatment (OR 3.38; 95% CI 1.34–8.56; *P* = 0.009) and biopsy-proven acute rejection (OR 5.05; 95% CI 1.16–21.90; *P* = 0.031) were independent risk factors, whereas low-dose Rit was not associated with neutropenia (OR 2.65; 95% CI 0.63–11.0; *P* = 0.18) (Table 4).

**Table 4:** Univariate and multivariate analysis of factors associated with grade 4 neutropenia

Variables	Univariate analysis			Multivariate analysis		
	HR	95% CI	*P-value*	HR	95% CI	*P-value*
Recipient age (year)	0.98	0.96—1.01	0.30			
Gender (female)	0.77	0.30—1.95	0.57			
BMI (kg/m2)	1.07	0.96—1.20	0.21			
ABO compatibility						
Minor-mismatched (ref. matched)	0.38	0.08—1.83	0.23			
Major-mismatched (ref. matched)	1.48	0.62—3.58	0.37			
Preformed DSA	0.80	0.28—2.31	0.68			
HTN	0.74	0.32—1.72	0.48			
DM	0.86	0.30—2.47	0.78			
PEKT	1.66	0.69—3.98	0.25			
Duration of dialysis (month)	0.99	0.99—1.00	0.54			
Initial MMF dose > 25 mg/kg	2.68	1.15—6.22	0.021	1.54	0.59—3.99	0.37
Low dose Rit	2.54	0.90—7.12	0.077	2.65	0.63—11.0	0.18
Plasmapheresis	1.97	0.84—4.63	0.11	1.11	0.34—3.66	0.86
Anti-CMV treatment	3.72	1.57—8.82	0.028	3.38	1.34—8.56	0.009
Biopsy proven acute rejection	9.29	2.42—35.7	0.001	5.05	1.16—21.9	0.031

*BMI* Body mass index, *DSA* Donor specific antibody, *HTN* Hypertension, *DM* Diabetes mellitus, *PEKT* Preemptive kidney transplantation, *MMF* Mycophenolate mofetil, *CMV* Cytomegalovirus

### PLS monitoring and peripheral B-lymphocyte

**Fig. 2:**
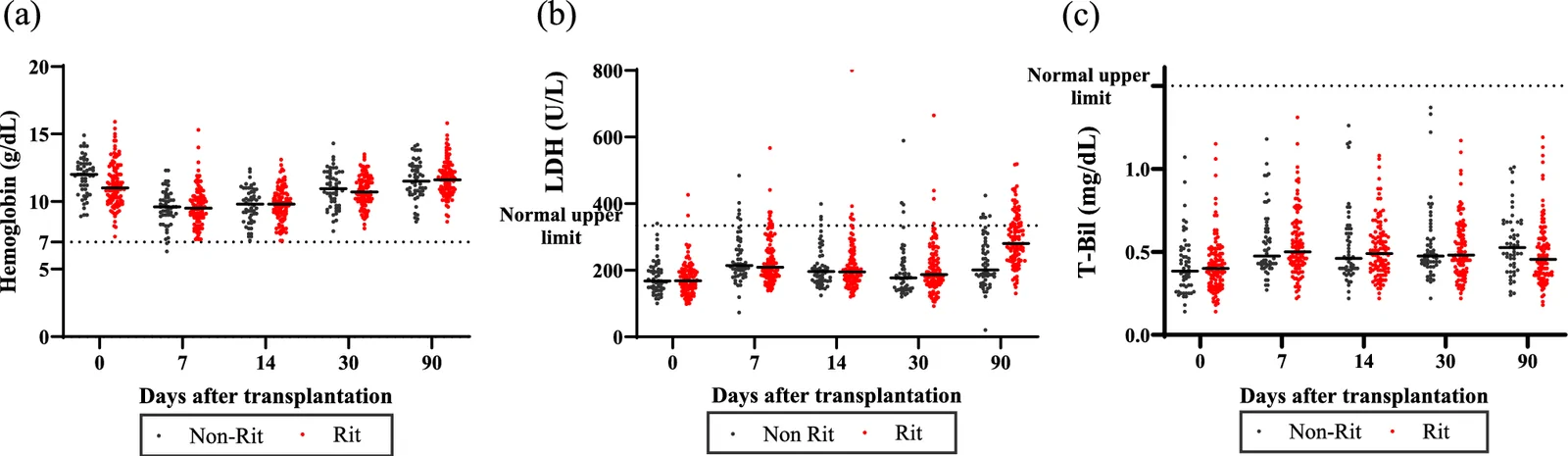
Perioperative transitions in laboratory parameters associated with passenger lymphocyte syndrome (PLS) **a** hemoglobin, **b** LDH, and **c** Total bilirubin (T-Bil)

Systematic assessment for PLS demonstrated that 10 recipients required blood transfusion due to hemoglobin decline after 1 week or more postoperatively: 4 (7.4%) in the Non-Rit group and 6 (5.6%) in the Rit group. However, in all transfused cases, haptoglobin was measured and found to be within the normal range (67–305 mg/dL), indicating no evidence of hemolytic anemia. Additionally, serial monitoring of LDH and T-bil did not reveal any patterns consistent with PLS in either group within 3 months (Fig. 2). At 1 year post-transplant, the median percentage of peripheral B-lymphocytes was significantly lower in the Rit group, 0% (IQR 0–2), compared to 8% (IQR 5–11) in the Non-Rit group (*P* < 0.001), confirming effective B-cell depletion (Table 3).

## Discussion

Our study demonstrates that all recipients achieved excellent 1 year graft survival rates of 100%, with comparable long-term outcomes despite the higher immunological risk profile in the Rit group. This finding supports the efficacy of our low-dose Rit protocol in managing immunologically high-risk KTx without compromising safety. Importantly, when considered within the broader landscape of desensitization strategies, the performance of low-dose Rit warrants recognition as an optimal therapeutic approach.

The immunological rationale for this success lies in Rit’s dual mechanism: (1) direct elimination of CD20 + B lymphocytes before engraftment, and (2) disruption of T-B cell crosstalk critical for antibody production [26–28]. Notably, median B-lymphocyte percentage remained significantly lower at 1 year, confirming persistent immunomodulation. While de novo DSA production was equivalent between groups, the median MFI was lower in the Rit group, suggesting attenuated antibody responses through sustained B-cell suppression. These findings validate our protocol adoption and align with the seminal study by Shirakawa et al., which demonstrated that 200 mg/body represents a sufficient pharmacodynamic threshold for CD19 + depletion in ABO-incompatible KTx [6].

While a systematic review identified a 20% PLS incidence in ABO-mismatched KTx, with 37.4% developing severe anemia (hemoglobin < 60 g/L) [16, 17], our comprehensive PLS monitoring, including haptoglobin assessment, revealed no evidence of hemolytic anemia in either group. This discrepancy underscores the limitations of traditional cyclosporine-based regimens compared to our tacrolimus-MMF-Rit. protocol, which provides more comprehensive B-cell suppression. Rit induces preoperative B-cell depletion, preventing the antigen presentation cascade that triggers PLS. The clinical significance of these finding warrants consideration in future desensitization protocols.

The safety of low-dose Rit with respect to infectious complications deserves particular emphasis given historical concerns about Rit-related immunosuppression. Nishida et al. compared 54 ABO-incompatible recipients receiving Rit (200 or 500 mg/body) with 29 control recipients, finding similar CMV infection rates in CMV-seropositive patients (58.1% vs. 75%, P = 0.1676) [7]. Notably, CMV-seronegative recipients receiving Rit developed seroconversion without severe disease, suggesting preserved CD8 + T-lymphocyte immunity. Several studies revealed that Rit preserves protective factors—including plasma cells, long-lived memory B cells, and splenic CD27 + memory B-cell niches—that do not express CD20 [29, 30]. These findings provide reassurance that Rit achieves selective B-cell targeting without broad immunological compromise. Although low-dose Rit was associated with Grade 4 neutropenia in univariate analysis, our multivariate analysis revealed that anti-CMV treatment and biopsy-proven acute rejection were the true independent risk factors, whereas low-dose Rit was not. This suggests that the higher crude neutropenia incidence in the Rit group was primarily driven by their need for these intensive interventions, indicating minimal direct impact from Rit itself. Furthermore, comparable infection rates (CMV, UTI, and fungal) across groups confirm that this protocol does not compound severe secondary complications despite the patients’ higher immunological risk. These finding directly contradicts concerns raised during the historical evaluation of Rit in transplantation and provide quantitative evidence of the safety profile of low-dose approaches. The combination of effective immunosuppression without excessive infection risk suggests broader applicability to marginal donors, potentially expanding Japan’s limited donor pool while maintaining excellent outcomes.

While emerging immunoabsorption and plasmapheresis techniques challenge Rit-based protocols, low-dose Rit offers distinct advantages. First, unlike antibody-removal approaches, Rit targets the B-cell source of antibody production, explaining durable DSA control. Second, selective CD20 + targeting preserves other adaptive immune compartments, distinguishing it from non-selective lymphocyte-depleting agents (rabbit antithymocyte globulin, murine antihuman CD3 antigen). Third, low-dose Rit avoids morbidity from graft irradiation and splenectomy. Our experience demonstrates that splenectomy-free ABO-incompatible transplantation is safely achievable without plasmapheresis in minor ABO-mismatched recipients—a substantial simplification compared to historical practice.

The present study had several limitations. First, the Rit group comprised higher-risk recipients (minor/major ABO-mismatches, DSA-positive, and FSGS patients), yet comparable graft survival rates provide substantial evidence of efficacy despite this bias toward the null hypothesis. Second, the heterogeneity of concurrent plasmapheresis precludes definitive attribution to Rit monotherapy versus combination approaches. Future randomized controlled trials should prospectively evaluate low-dose Rit in standardized subgroups (ABO-mismatched, HLA-sensitized) with parallel comparison arms. Furthermore, while the sensitive MFI cutoff of > 500 for DSA positivity was consistently utilized over the 15 years, potential lot-to-lot variations in the Luminex assays during this extended period cannot be entirely ruled out. Third, whether extended B-cell suppression (median 0% at 1 year) represents therapeutic benefit or potential liability remains undefined. Long-term studies evaluating vaccine responses, thymic T-cell production, and pathogen-specific immune memory are needed to determine whether brief pre-transplant rituximab establishes sustained beneficial immunomodulation or risks late complications.

## Conclusion

In conclusion, this 15-year cohort demonstrates that low-dose Rit represents an effective and safe desensitization strategy. By achieving excellent 1-year graft and patient survival rates of 100%, preventing PLS through comprehensive monitoring, and demonstrating favorable safety profiles including minimal impact on severe neutropenia risk, this protocol offers a practical approach to managing immunologically high-risk kidney transplant recipients while potentially expanding the donor pool. 

## Supplementary Information

Below is the link to the electronic supplementary material.

Supplementary file1 (PDF 219 KB)

## Abbreviations

BMI: Body mass index
CMV: Cytomegalovirus
CTCAE: Common terminology criteria for adverse events
DAT: Direct antiglobulin testing
DFPP: Double-filtration plasmapheresis
DSA: Donor-specific antibody
ESKD: End-stage kidney disease
FSGS: Focal segmental glomerulosclerosis
G-CSF: Granulocyte colony-stimulating factor
HLAi: Human leukocyte antigen antibody-incompatible
IQR: Interquartile range
IVIG: Intravenous immunoglobulin
KTx: Kidney transplantation
LDH: Lactate dehydrogenase
MFI: Mean fluorescent index
MMF: Mycophenolate mofetil
PEX: Plasma exchange
PLS: Passenger lymphocyte syndrome
Rit: Rituximab
T-bil: Total bilirubin
UTI: Urinary tract infection

## References

[1] Abecassis M, Bartlett ST, Collins AJ, et al. Kidney transplantation as primary therapy for end-stage renal disease: a National Kidney Foundation/Kidney Disease Outcomes Quality Initiative (NKF/KDOQI™) conference. Clin J Am Soc Nephrol. 2008;3(2):471–80. 18256371 10.2215/CJN.05021107 PMC2390948

[2] de Brito DCS, Machado EL, Reis IA, et al. Modality transition on renal replacement therapy and quality of life of patients: a 10-year follow-up cohort study. Qual Life Res. 2019;28(6):1485–95. 30666548 10.1007/s11136-019-02113-z

[3] Inui M, Ishida H, Omoto K, et al. Kidney transplantation at Tokyo Women’s Medical University. Clin Transpl. 2011:127–43. 22755409

[4] Ko Y, Kim JY, Kim SH, et al. Acute rejection and infectious complications in ABO- and HLA-incompatible kidney transplantations. Ann Transplant. 2020;25:e927420. 33020465 10.12659/AOT.927420 PMC7547531

[5] Montgomery RA, Lonze BE, King KE, et al. Desensitization in HLA-incompatible kidney recipients and survival. N Engl J Med. 2011;365(4):318–26. 21793744 10.1056/NEJMoa1012376

[6] Shirakawa H, Ishida H, Shimizu T, et al. The low dose of rituximab in ABO-incompatible kidney transplantation without a splenectomy: a single-center experience. Clin Transplant. 2011;25(6):878–84. 21175849 10.1111/j.1399-0012.2010.01384.x

[7] Nishida H, Ishida H, Tanaka T, et al. Cytomegalovirus infection following renal transplantation in patients administered low-dose rituximab induction therapy. Transpl Int. 2009;22(10):961–9. 19619177 10.1111/j.1432-2277.2009.00903.x

[8] Hirai T, Ishida H, Toki D, et al. Comparison of the acute rejection incidence rate in spousal donor transplantation before and after anti-CD20 antibody (Rituximab) protocol as desensitization therapy. Ther Apher Dial. 2011;15(1):89–97. 21272258 10.1111/j.1744-9987.2010.00856.x

[9] Takahashi K, Saito K, Takahara S, et al. Results of a multicenter prospective clinical study in Japan for evaluating efficacy and safety of desensitization protocol based on rituximab in ABO-incompatible kidney transplantation. Clin Exp Nephrol. 2017;21(4):705–13. 27534951 10.1007/s10157-016-1321-5

[10] Macklin PS, Morris PJ, Knight SR. A systematic review of the use of Rituximab for desensitization in renal transplantation. Transplant. 2014;98(8):794–805. 10.1097/TP.0000000000000362 25321163

[11] Barnett ANR, Hadjianastassiou VG, Mamode N. Rituximab in renal transplantation. Transpl Int. 2013;26(6):563–75. 23414100 10.1111/tri.12072

[12] Yoshinaga K, Araki M, Wada K, et al. Low-dose rituximab induction therapy is effective in immunological high-risk renal transplantation without increasing cytomegalovirus infection. Int J Urol. 2020;27(12):1136–42. 33012030 10.1111/iju.14382

[13] Lee JH, Lee HR, Lee SW, et al. Effect of induction therapy dose on survival in ABO-incompatible kidney transplantation: A network meta-analysis using recent data. Transplant Proc. 2024;56(3):511–4. 38378338 10.1016/j.transproceed.2024.01.026

[14] Tomita Y, Iwadoh K, Ogawa Y, et al. Single fixed low-dose rituximab as induction therapy suppresses de novo donor-specific anti-HLA antibody production in ABO compatible living kidney transplant recipients. PLoS ONE. 2019;14(10):e0224203. 31644555 10.1371/journal.pone.0224203 PMC6808551

[15] Araki M, Wada K, Mitsui Y, et al. The efficacy of rituximab in high-risk renal transplant recipients. Acta Med Okayama. 2016;70:295–7. 27549676 10.18926/AMO/54507

[16] Moosavi MM, Duncan A, Stowell SR, et al. Passenger lymphocyte syndrome; a review of the diagnosis, treatment, and proposed detection protocol. Transfus Med Rev. 2020;34(3):178–87. 32826130 10.1016/j.tmrv.2020.06.004

[17] Zhao H, Ding Z, Luo Z, et al. Passenger lymphocyte syndrome in renal transplantation: a systematic review of published case reports. Transpl Immunol. 2022;73:101605. 35487476 10.1016/j.trim.2022.101605

[18] Mathavan A, Krekora U, Kleehammer AC, et al. Passenger lymphocyte syndrome following minor ABO-mismatched liver transplantation. BMJ Case Rep. 2024;17(3):e259259. 38453222 10.1136/bcr-2023-259259 PMC10921429

[19] Yeom GE, Lim SH, Kim JH, et al. Gastrointestinal involvement of passenger lymphocyte syndrome followed by minor ABO-incompatible renal transplantation: A case report. Pediatr Transplant. 2023;27(6):e14556. 37300335 10.1111/petr.14556

[20] Sokol RJ, Stamps R, Booker DJ, et al. Posttransplant immune-mediated hemolysis. Transfusion. 2002;42(2):198–204. 11896335 10.1046/j.1537-2995.2002.00026.x

[21] Ishida H, Tanabe K, Tokumoto T, et al. The evaluation of graft irradiation as a method of preventing hemolysis after ABO-mismatched renal transplantation. Transpl Int. 2002;15(8):421–4. 12221462 10.1007/s00147-002-0442-9

[22] Ishida H, Inui M, Furusawa M, et al. Late-onset neutropenia (LON) after low-dose Rituximab treatment in living related kidney transplantation--single-center study. Transpl Immunol. 2013;28(2–3):93–9. 23353568 10.1016/j.trim.2013.01.003

[23] Nishide S, Uchida J, Kaei K, et al. Passenger lymphocyte syndrome in the ABO-incompatible kidney transplant recipient receiving Rituximab. Exp Clin Transplant. 2019;17(4):456–9. 10.6002/ect.2016.0261 28664822

[24] Tsujimura K, Ishida H, Tanabe K. Is efficacy of the anti-CD20 antibody Rituximab preventing hemolysis due to passenger lymphocyte syndrome? Ther Apher Dial. 2017;21(1):22–5. 27786418 10.1111/1744-9987.12483

[25] Kanda Y. Investigation of the freely available easy-to-use software “EZR” for medical statistics. Bone Marrow Transplant. 2013;48(3):452–8. 23208313 10.1038/bmt.2012.244 PMC3590441

[26] Marino M, Bartoccioni E, Alboini PE, et al. Rituximab in myasthenia gravis: a “to be or not to be” inhibitor of T cell function. Ann N Y Acad Sci. 2018;1413(1):41–8. 29369382 10.1111/nyas.13562

[27] Kessel A, Rosner I, Toubi E. Rituximab: beyond simple B cell depletion. Clin Rev Allergy Immunol. 2008;34(1):74–9. 18240027 10.1007/s12016-008-8074-1

[28] Kamburova EG, Koenen HJPM, Boon L, et al. In vitro effects of rituximab on the proliferation, activation and differentiation of human B cells. Am J Transplant. 2012;12(2):341–50. 22070501 10.1111/j.1600-6143.2011.03833.x

[29] Ramos EJ, Pollinger HS, Stegall MD, et al. The effect of desensitization protocols on human splenic B-cell populations in vivo. Am J Transplant. 2007;7(2):402–7. 17241113 10.1111/j.1600-6143.2006.01632.x

[30] Agarwal A, Vieira CA, Book BK, et al. Rituximab, anti-CD20, induces in vivo cytokine release but does not impair ex vivo T-cell responses. Am J Transplant. 2004;4(8):1357–60. 15268740 10.1111/j.1600-6143.2004.00502.x

