# Supplementary material for: Optimizing low-dose rituximab protocol for ABO-mismatched kidney transplantation: long-term outcomes in a single-center retrospective cohort study
Source: Clin Exp Nephrol. 2026 May 26;30(9):1359–67. doi: 10.1007/s10157-026-02882-1 (PMC13518381; doi:10.1007/s10157-026-02882-1)
Supplement: Supplementary file 1 [file 10157_2026_2882_MOESM1_ESM.pdf]

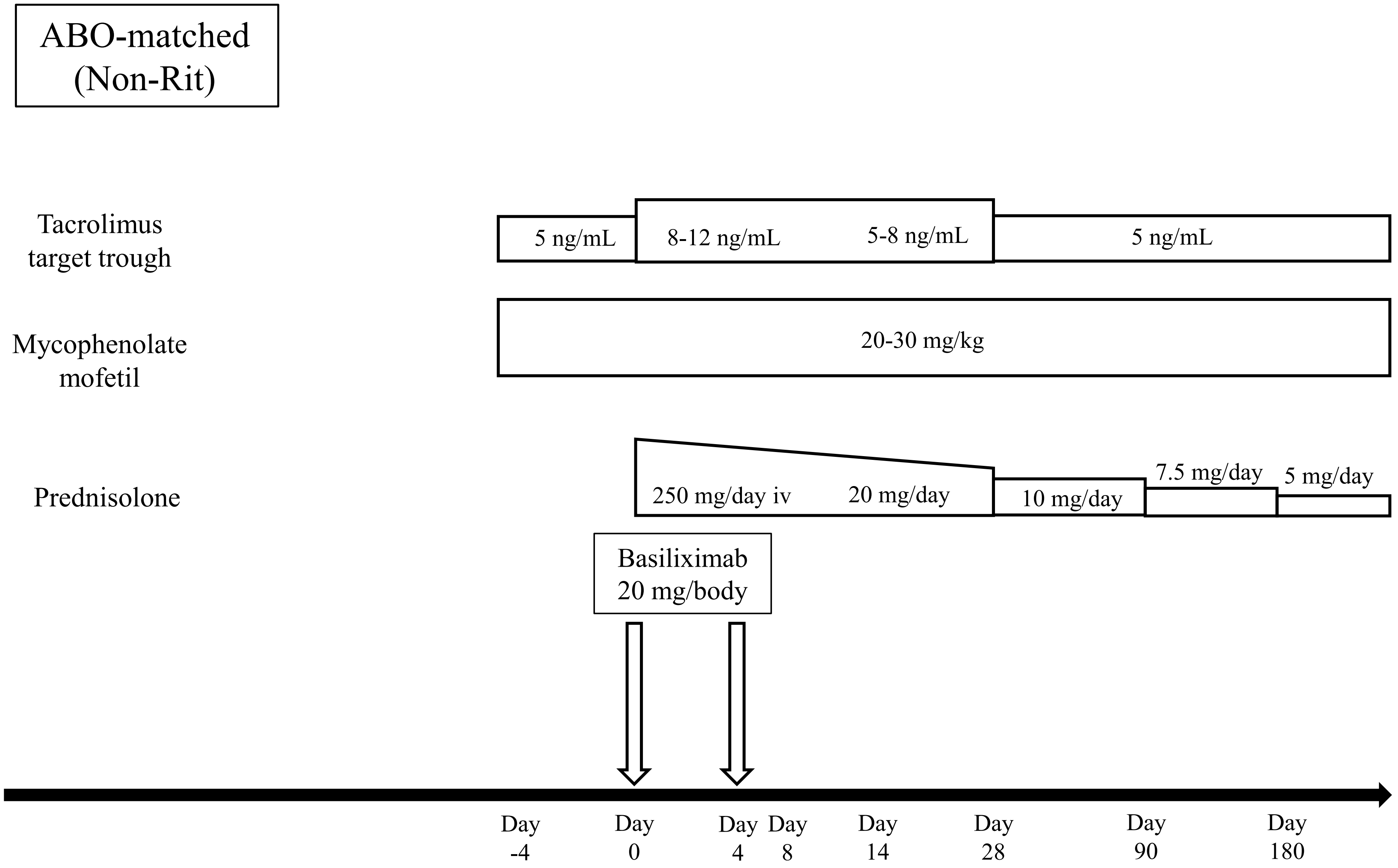

Figure S1. Immunosuppression protocol for recipients in the Non-Rit group.

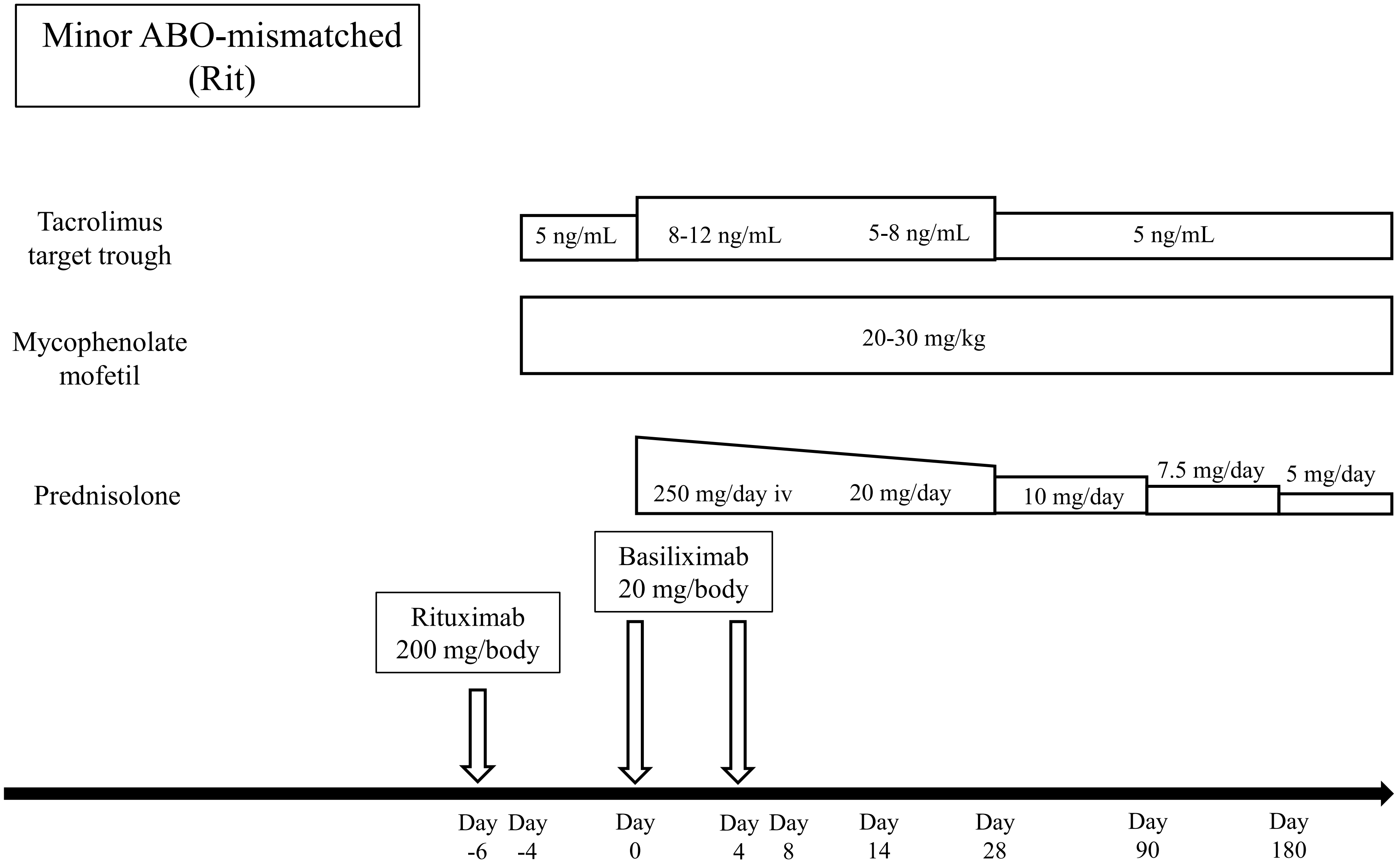

Figure S2. Immunosuppression protocol for recipients in the Rit group with minor ABO-mismatch.

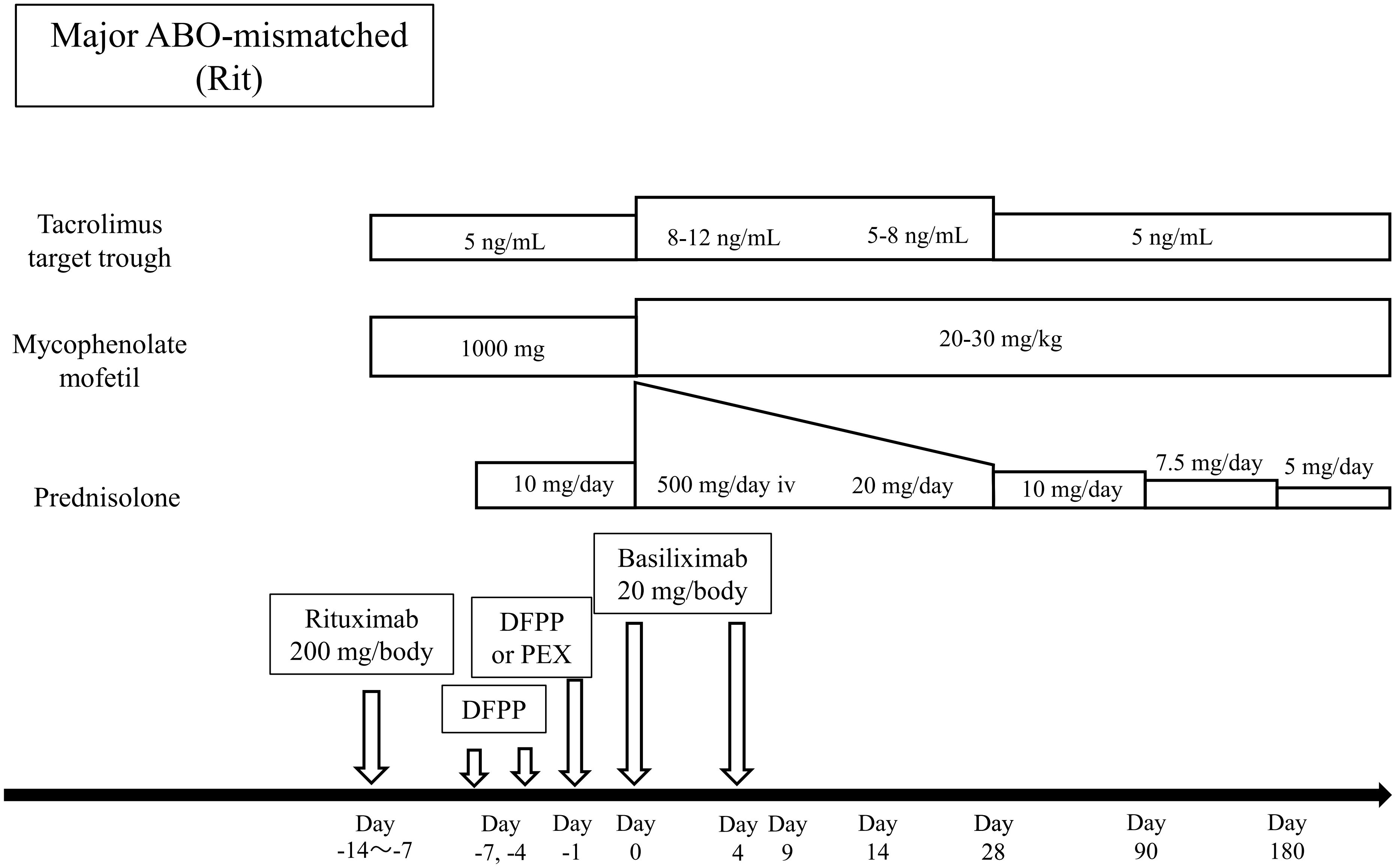

Figure S3. Immunosuppression protocol for recipients in the Rit group with major ABO- mismatch.

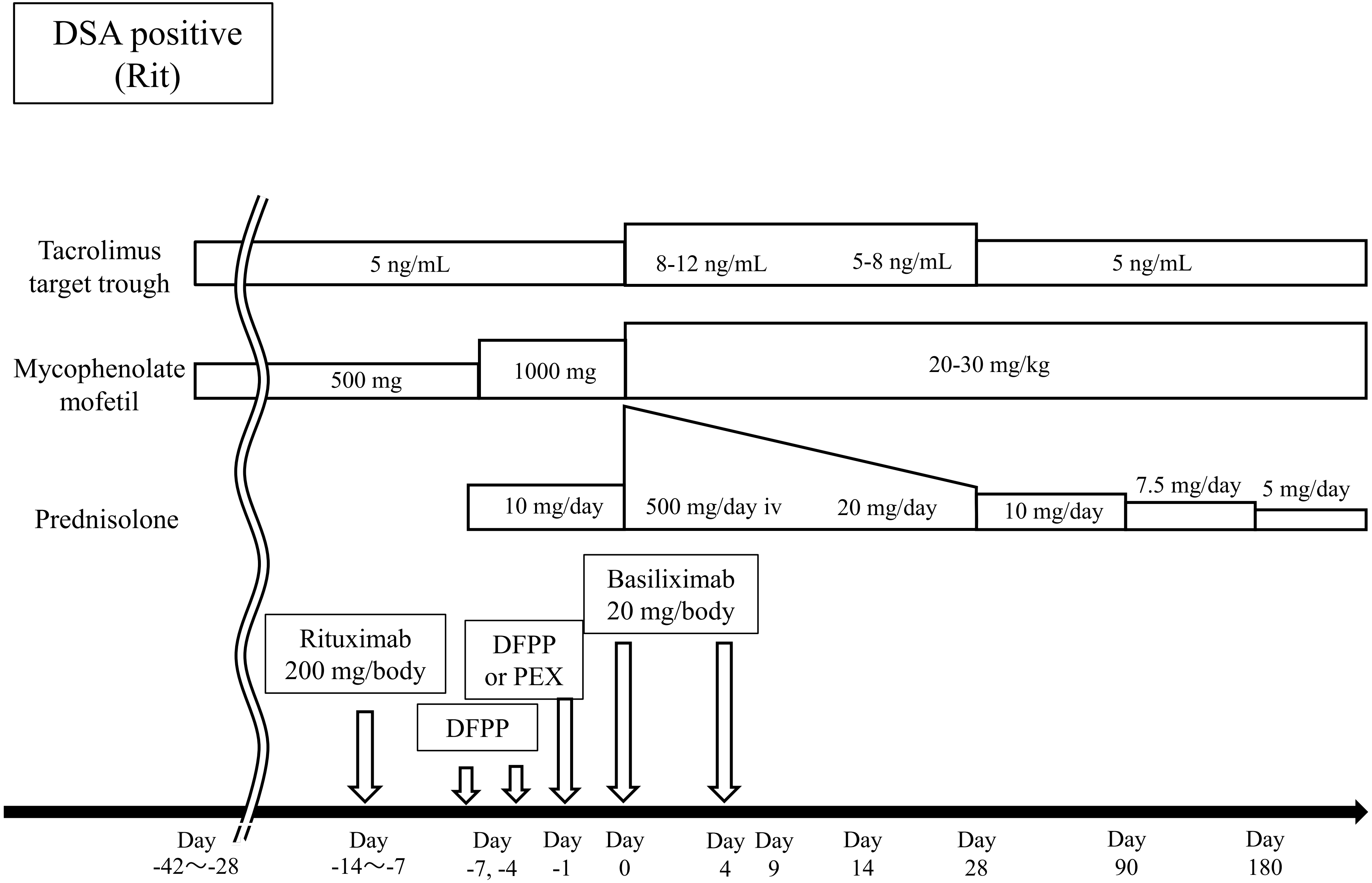

Figure S4. Immunosuppression protocol for recipients in the Rit group with DSA-positive.

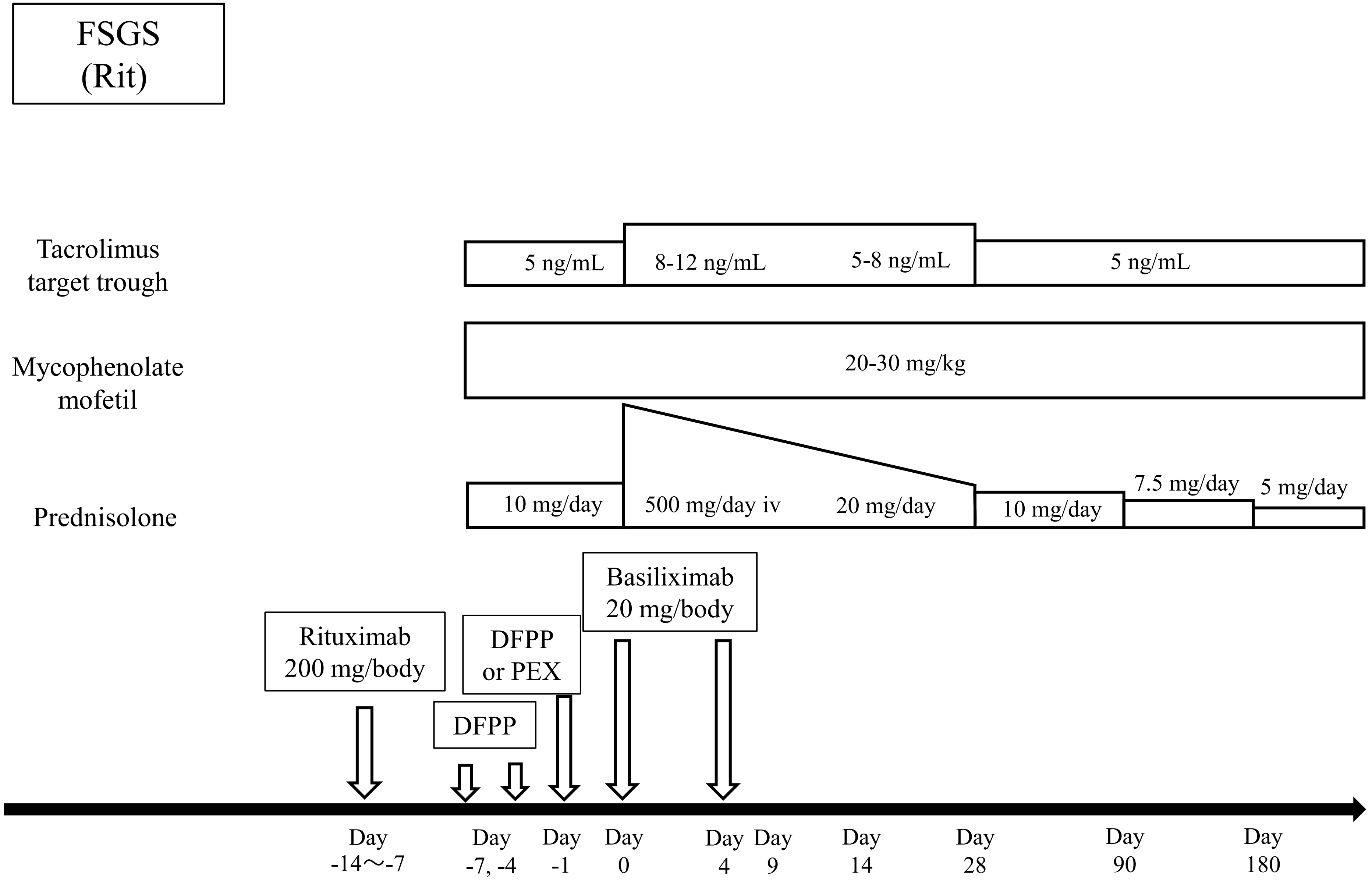

Figure S5. Immunosuppression protocol for recipients in the Rit group with FSGS.
